# Restricted Surface Diffusion of Cytochromes on Bioenergetic Membranes with Anionic Lipids

**DOI:** 10.3390/membranes15040124

**Published:** 2025-04-13

**Authors:** Aaron Chan, Emad Tajkhorshid

**Affiliations:** 1 Theoretical and Computational Biophysics Group, NIH Resource for Macromolecular Modeling and Visualization, Beckman Institute for Advanced Science and Technology, University of Illinois Urbana, Champaign, IL 61801, USA; aaronnc2@illinois.edu; 2 Center for Biophysics and Quantitative Biology, University of Illinois Urbana, Champaign, IL 61801, USA

**Keywords:** bioenergetic membranes, electron transfer, enhanced sampling, cardiolipin, molecular dynamics

## Abstract

Bioenergetic membranes of mitochondria, thylakoids, and chromatophores are primary sites of ATP production in living cells. These membranes contain an electron transport chain (ETC) in which electrons are shuttled between a series of redox proteins during the generation of ATP via oxidative phosphorylation. The phospholipid composition of these membranes, which often include negative lipids, plays a role in determining the electrostatics of their surface owing to the spatial distribution of their charged head groups. Cardiolipin (CDL) is a phospholipid commonly associated with bioenergetic membranes and is also a significant contributor to the negative surface charge. Interactions between cytochromes and phospholipid head groups in the membrane can in principle affect the rate of its travel between ETC components, hence influencing the rate of ATP turnover. Here, we use molecular dynamic (MD) simulations that feature an accelerated membrane model, termed highly mobile membrane mimetic (HMMM), to study protein–lipid interactions during the diffusion of cytochrome *c*_2_ between redox partners in a bioenergetic membrane. We observe a “skipping” mode of diffusion for cytochromes along with a bias for binding to anionic lipids, particularly with a strong preference for CDL. During diffusion, cytochrome *c*_2_ maintains a relatively fixed tilt with respect to the membrane normal with wider fluctuations in its angle with respect to the plane of the membrane. The obtained results describing the behavior of cytochrome *c*_2_ on a representative bioenergetic membrane have direct ramifications in shuttling motions of other similar electron-carrying elements in other bioenergetic membranes, which are composed of a significant amount of anionic lipids. The mode of surface-restricted diffusion reported here would modulate rapid electron transfer between the ETC complexes anchored in bioenergetic membranes by reducing the search space between them.

## 1. Introduction

**Figure 1:**
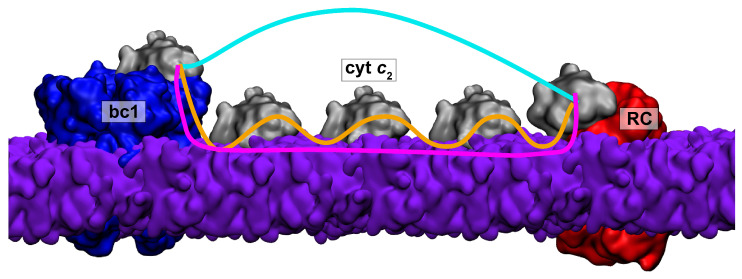
Potential modes of cytochrome ***c*****_2_** diffusion on a bioenergetic membrane. Three routes of diffusion for cyt c_2_ are considered: through the solvent (cyan), hopping between membrane proteins and the membrane (orange), and sliding on the membrane surface (magenta).

Bioenergetic membranes are biological membranes associated the production of ATP. Examples of bioenergetic membranes include mitochondrial membranes in animal cells, thylakoid membranes in plants, and chromatophores in bacteria. A characteristic feature of bioenergetic membranes is the presence of an electron transport chain (ETC). An ETC can be described as a series of components between which electrons are transferred through a chain of donors and acceptors via redox reactions; the energy of electron transfer in every step is converted to a form of chemical energy, usually an electrochemical gradient across the membrane, which can be used by the cell. The end result of these redox reactions is a proton gradient that drives ATP synthesis through the membrane-bound enzyme ATP synthase. Cytochromes play important roles in ETCs by shuttling electrons between different components (Figure 1) [1].

Cytochrome *c*_2_ (cyt *c*_2_) is a cytochrome associated with the photosynthetic pathway of *R. sphaeroides* [2,3]. A homologous protein, cytochrome *c*, has an analogous function in aerobic respiration and is found in the intermembrane space of mitochondria. The rate at which cyt *c*_2_ diffuses from one ETC complex to the next is expected have a significant influence on the rate of ATP production; it occurs over larger spatial scales than intra-protein charge transfer processes [4,5,6].

**Figure 2:**
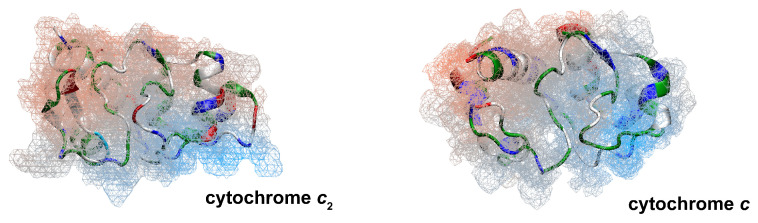
Polarity of ETC electron shuttling proteins. Proteins that act as intercomplex electron shuttles, e.g., cyt *c*_2_ and cyt *c*, typically feature a polarized surface. Proteins are colored according to residue type (blue for positive/basic, red for negative/acidic, white for nonpolar/hydrophobic, and green for polar/neutral). The electrostatic potential maps are represented with meshes.

Proteins that act as electron shuttles contain a notable degree of electrostatic polarity in their structure; these proteins often contain a concentrated patch of charged amino acids at a surface face [7,8,9,10,11,12,13,14,15]. Examples of this can be seen in the structures of cytochrome *c*_2_ and cytochrome *c*, as shown in Figure 2. The polar nature of the cytochrome surfaces suggests that the travel between ETC complexes can be influenced by the electrostatics of proteins and lipid bilayers.

Cardiolipin (CDL) is an anionic lipid with a conical geometry, often present in bioenergetic membranes associated with ETCs [16]. The ability of CDL to induce curvatures has been shown to influence the assembly of supercomplexes within the ETCs of bioenergetic membranes [17,18]; the associated change in the surface area of a membrane can change the effective search distance between binding partners. Previous studies have also suggested that charged lipids, e.g., CDL and phosphatidylglycerol (PG), can influence the mode of cytochrome diffusion; however, the effects of lipid heterogeneity and path of diffusion are not well understood [19,20,21,22]. In this study, we aim to elucidate the role of lipid composition in cytochrome binding and diffusion in the context of traversing a heterogeneous anionic lipid bilayer.

## 2. Methods

### 2.1. HMMM Simulation System

Molecular dynamic (MD) simulations using a high-mobility membrane mimetic (HMMM) [23] were used to probe the diffusion of cyt *c*_2_ (PDB 1L9B) [9] on the surface of a lipid bilayer. The HMMM model was developed as a membrane model that balances computational efficiency with atomic-level detail where it is most needed, namely at the membrane surface [23,24,25]. The model approximates the hydrophobic core of the lipid bilayer by replacing a portion of the phospholipid tails with organic solvents such as 1,1-dichloroethane (DCLE) or short-chain alkanes [23,24,25]. This modification significantly reduces the membrane viscosity and enhances lateral lipid diffusion, thereby markedly accelerating membrane dynamics [23]. By facilitating faster lipid movements and increasing the accessibility of the membrane surface, the HMMM model enhances the rate of membrane-associated reactions, such as the binding and unbinding of peripheral proteins [26]. Importantly, it does so without compromising the atomistic representations of the lipid head groups, allowing for more accurate modeling of interfacial effects such as hydrogen bonds, hydration, and specific lipid–protein interactions [23].

**Figure 3:**
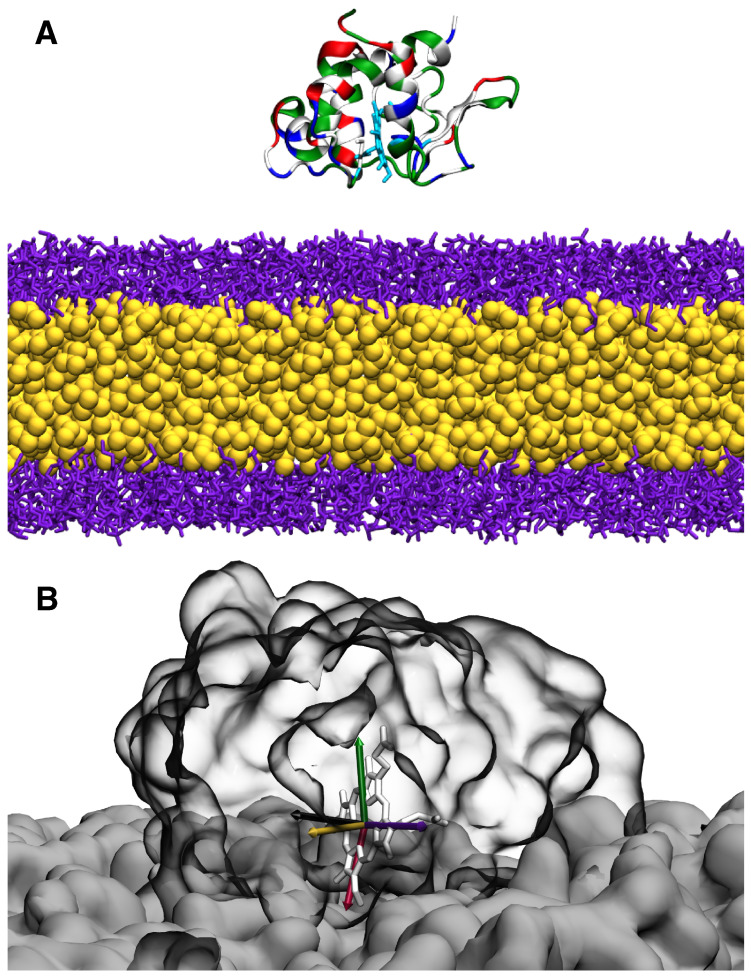
HMMM model configuration of cyt *c*_2_ lipid diffusion. A diagram of a representative simulation system is presented in (**A**). Cytochrome *c*_2_ is initially placed 40 Å above the center of the HMMM membrane. The membrane comprises truncated lipids (purple) and the solvent 1,2-dichloroethane (DCLE, yellow). The protein is colored according to residue type (blue, positive/basic; red, negative/acidic; green, polar/non-charged; and white, hydrophobic). The set of Euler angles used to define the geometric orientation of cyt *c*_2_ are presented in (**B**). Legend: $\Phi_{\beta }$ (green:black), $\Phi_{\alpha }$ (purple:yellow), and $\Phi_{\gamma }$ (purple:red).

The phospholipid composition of the chromatophore in *R. sphaeroides* was used for the simulations [27]. The lipid composition for the bilayer used in our study was 14% CDL, 30% phosphatidylethanolamine (PE), 46% phosphatidylglycerol (PG), and 10% phosphatidylcholine (PC). The membranes were generated using CHARMM-GUI [28,29] with the center of the bilayer as the origin of the system. The protein was initially placed in the solution with the positively charged face oriented towards the binding leaflet of the membrane, with its center of mass 40 Å above the midpoint of the bilayer, thereby ensuring that no initial contacts between the protein and the membrane existed. The system was solvated with 0.15 M KCl and the TIP3P water model [30] in a (110 Å × 110 Å × 120 Å) unit cell using VMD [31]. The system setup is shown in Figure 3.

All simulations were performed using NAMD [32] with the CHARMM36 force field and the CHARMM36m refinement [33,34]. Parameters for the porphyrin ring of heme were adopted from previous studies [3,35]. All simulations in this study were equilibrated as NPT ensembles, with T=310 K, and P=1.0 atm using Langevin-based temperature and pressure controls [36,37]. A multi-step equilibration scheme was used to allow the system to reach equilibrium; the spring constants for the harmonic restraints on the lipid head groups, protein backbone, and lipid tails were decreased gradually from 2.5 to 0.0 kcal·mol^−1^·Å^−2^. Harmonic restraints with a spring constant of 0.05 kcal·mol^−1^·Å^−2^ acting in the *z* direction were maintained on the carbonyl groups of the short-tailed phospholipids during the production runs of the HMMM systems. The production dataset consists of ten independently generated replicates; each of the replicates were ran for 200 ns using 2.0 fs timesteps on NCSA Blue Waters. HMMM simulation results were validated by full-membrane MD simulations, continued from HMMM simulations after conversion, to verify that the use of the HMMM approximate model did not bias the observations. The full-membrane continuation MD simulations were ran for 100 ns using the same parameters as the HMMM dataset, but without any restraints on the lipids after equilibration.

### 2.2. Characterization of the Membrane-Bound State of *cyt* c_*2*_

The membrane-bound state of cyt *c*_2_ was characterized in terms of its insertion depth and angular orientation. Membrane insertion depth was measured as the *z*-displacement of the center of mass for the side chain containing the atom furthest below the plane defined by the average *z*-value for the phosphate plane in the binding leaflet; if no protein residue penetrated the plane, the closest residue to the membrane was used to determine the value.

The orientation of cyt *c*_2_ is characterized by the tilt angles defined by the plane of the porphyrin ring of cyt *c*_2_ and the membrane normal vector; these angle definitions are as follows Figure 3B:

- $\Phi_{\beta }$: Angle made by the membrane normal (*z*-axis) and normal axis of the heme ring.
- $\Phi_{\gamma }$: Angle made by the membrane and heme co-normal with the vector in the plane of the heme ring.
- $\Phi_{\alpha }$: Angle made by the membrane and heme co-normal with the *x*-axis.

### 2.3. Protein–Lipid Interactions During *cyt* c_*2*_ Diffusion

The number of contacts between the proteins and lipids was analyzed over the course of the simulations. A residue is defined to be interacting with the membrane if it has at least one atom within 2.5 Å of either a phosphate or nitrogen of a phospholipid head group (lipid–protein coordination), or if it has an atom below the carbonyl group of a phospholipid tail (hydrophobic insertion). A spectrum of protein–lipid interactions was obtained from the trajectories to probe for preference to bind particular lipid types. The relative lipid affinity is quantified by the fractional contribution of lipid–protein interactions normalized to the lipid ratio for each lipid type in the bilayer. The fractional contribution of each lipid species were determined from the HMMM simulations, quantified as the fraction of the trajectory in which protein–lipid interactions could be attributed to that lipid species.

### 2.4. Calculation of *cyt* c_*2*_ Diffusion Coefficient

The diffusion coefficient was calculated from the mean square displacement (MSD) of the center of mass of cyt *c*_2_ relative to its initial position over 10 independent simulations, assuming the diffusion of cyt *c*_2_ can be approximated with a Fickian (non-anomalous) model of diffusion. The equation for model fitting used here was $<x^{2}>=2NDt$, where $<x^{2}>$ is the MSD for diffusion in *N* dimensions, with a diffusion coefficient *D*, as a function of time (*t*).

## 3. Results

**Figure 4:**
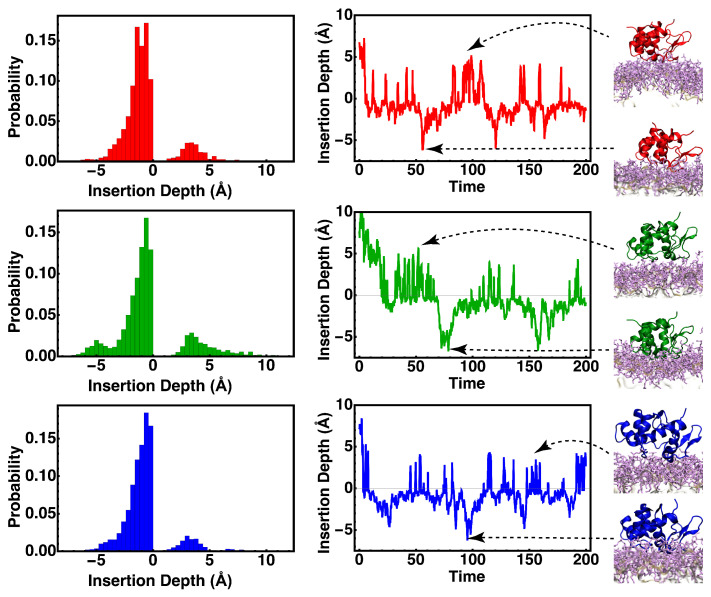
Membrane penetration of cyt *c*_2_. The insertion depth of cyt *c*_2_ is measured with respect to the phosphate plane of the binding leaflet. The time series for three replicates (**right**) and corresponding histogram (**left**) along with snapshots of the cyt *c*_2_ configuration relative to the membrane. Distinctive intervals in the time series coupled with the bimodal distribution of penetration depths suggest a “skipping” mode for the cyt *c*_2_ motion.

The lipid insertion depth measurements for three simulation replicates are shown in Figure 4. The presence of two peaks in the insertion depth distributions across replicates suggests that cyt *c*_2_ has at least two preferred membrane association modes. The time series for insertion depth suggests that cyt *c*_2_ does not penetrate the lipid very deeply but glides slightly above the membrane surface with transient insertions into the membrane, reminiscent of a skipping motion. The skipping mode of diffusion described in this study refers to two-dimensional (or restricted three-dimensional) diffusion on the surface of the membrane interspersed with short periods of three-dimensional diffusion or jumps. This mode of travel is supported by local maxima at −1 Å and 4 Å in the associated histogram set. (Figure 4). The two peaks in the histograms, which are also present when we analyze the trajectories individually (new Figure 4), represent two modes of interaction between cyt *c*_2_ and the membrane, one fully inserted (insertion depth < 0) and the other one on the surface of the membrane and likely contributing to the skipping motion of the protein.

**Figure 5:**
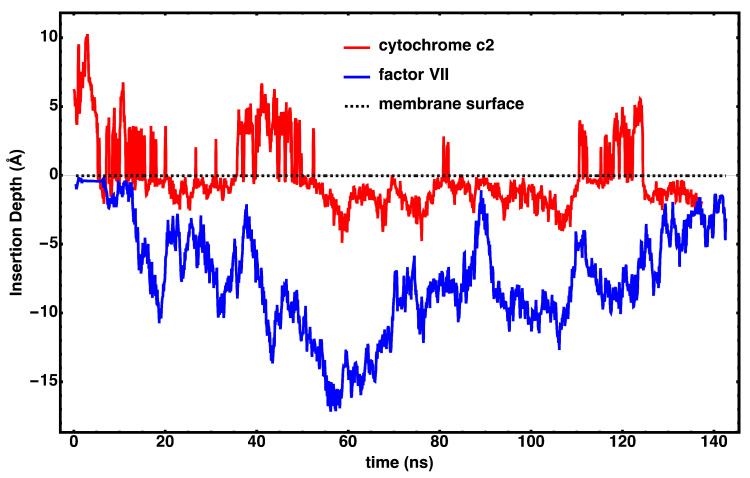
Characterizing the diffusion mode of cyt *c*_2_. The insertion depths for cyt *c*_2_ and Factor VII are compared. Insertion depth is measured with respect to the phosphate plane of the binding leaflet. Factor VII is associated with a mode of diffusion where it travels through the bilayer. In contrast, the time series for cyt *c*_2_ suggests only transient insertion during its migration. Videos for the trajectories of Factor VII and cyt *c*_2_ are available as SI Movies 1 and 2, respectively. Movies are shown only for the portion of the trajectory after the protein has arrived on the membrane surface.

The diffusion of cyt *c*_2_ is compared with that of the binding domain for Factor VII (Figure 5) [38]. Factor VII is a soluble protein associated with the coagulation cascade, and it is known to bind and diffuse through the membrane rather than skip across it [38]. Videos for the trajectories of Factor VII and cyt *c*_2_ are available as SI Movies 1 and 2, respectively. In contrast to Factor VII, which remains anchored into the membrane during the transition, cyt *c*_2_ is observed to travel on and off the membrane surface throughout the simulations.

**Figure 6:**
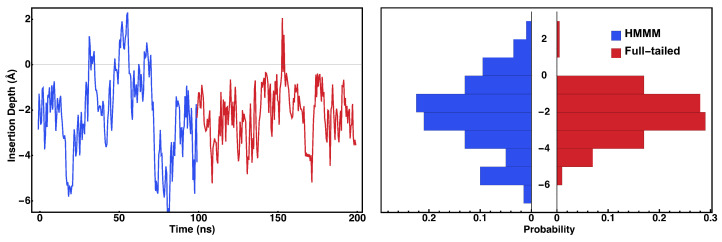
Validation of HMMM simulations with a full-membrane MD simulation. The distribution (**top**) and time series (**bottom**) for the insertion depth of cyt *c*_2_ are shown. The comparison between the HMMM simulation (blue) and its full-tailed continuation (red) suggests convergence of the HMMM simulations. Videos corresponding to trajectories shown are available as SI Movies 3 and 4.

The HMMM simulations were cross-validated by a conversion to a full-tailed membrane system and a subsequent 100 ns MD extension, as shown in Figure 6. Videos corresponding to trajectories, which are shown in Figure 6, are available as SI Movies 3 and 4. The insertion depths for the HMMM and full-tailed simulations are in agreement; the shift in the corresponding histograms can be attributed to the enhanced sampling rate of HMMM.

**Figure 7:**
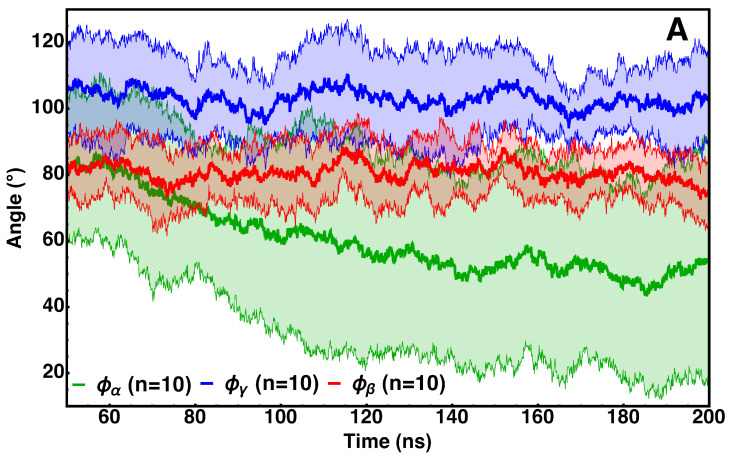

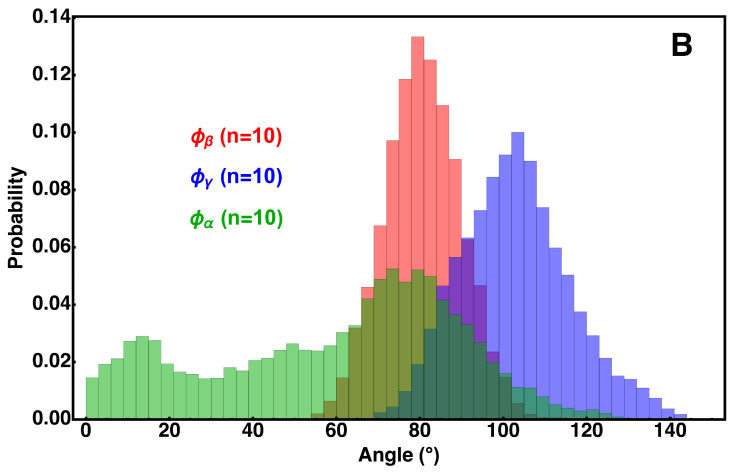
Membrane-bound orientation of cytochrome *c*_2_. The orientation of cyt *c*_2_ is defined through Euler angles using the simulation coordinate system as the original reference frame and the coordinate system of the heme ring for the rotated frame (see text for details). Time series for orientation angles averaged over ten independent trajectories are presented in (**A**). The corresponding histograms for the tilt angles are shown in (**B**). The preferred orientation of cyt *c*_2_ $\Phi_{\beta }=72.2^{\circ }$, $\Phi_{\alpha }=77.5^{\circ }$, and $\Phi_{\gamma }=101.1^{\circ }$.

The lipid-bound orientations of the protein and the distributions for the defined tilt angles are shown in Figure 7. The most likely orientations of cyt *c*_2_ ($\Phi_{\beta }=72.2^{\circ }$, $\Phi_{\alpha }=77.5^{\circ }$, and $\Phi_{\gamma }=101.1^{\circ }$) are shown in Figure 3B. We observed a finer spread for $\Phi_{\beta }$ in comparison to the other two tilt angles. This is within our expectations since tilting in the *z*-axis would move the negatively charged face of the cytochrome towards the negatively charged phosphate head groups of the lipids. We speculate that this may be a means to keep cyt *c*_2_ from entering an unfavorable conformation during travel between ETC complexes. These observations along with the corresponding time series measurements shown in Figure 7A suggest that cyt *c*_2_ remains in an upright orientation while traversing the membrane between ETC components.

**Figure 8:**
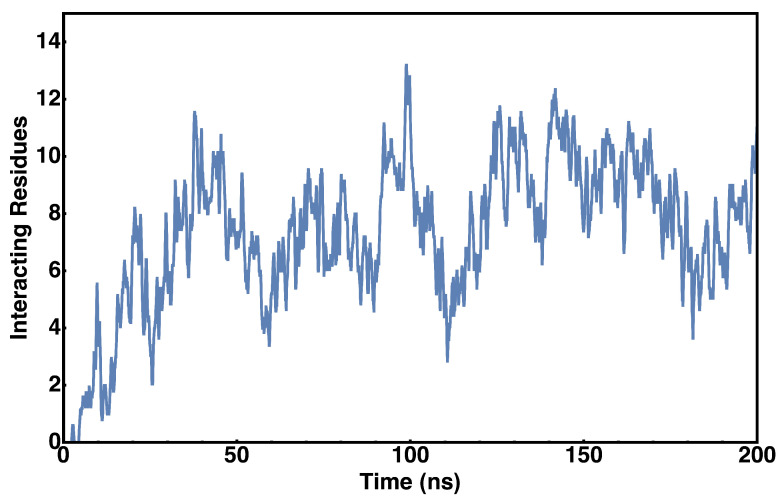

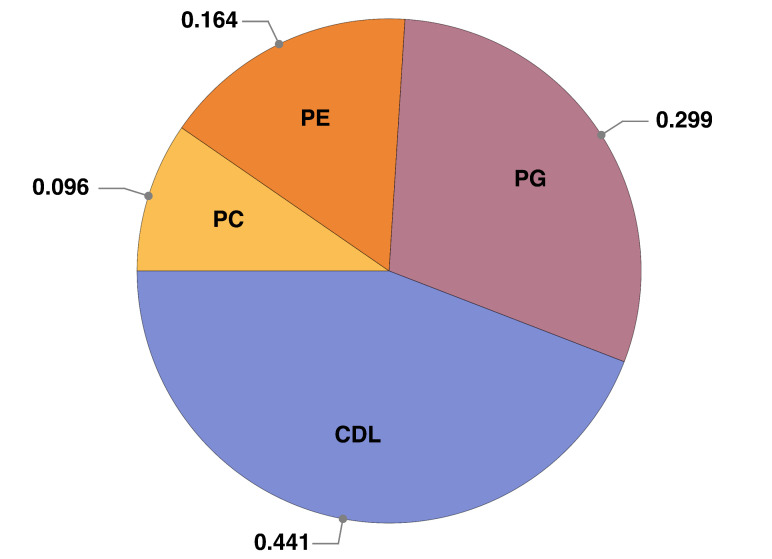
Cytochrome *c*_2_–lipid coordination. The time evolution for protein–lipid interactions during the diffusion of cyt *c*_2_ is shown (**top**). Interactions include both polar contacts with lipid head groups and hydrophobic insertions; the number of interacting residues does not discriminate between these modes of interactions. The pie chart (**bottom**) shows the fractional contribution of each lipid type over the course of the trajectory. Upon binding, which occurs after about 40 ns on average, we observe a mean number of eight lipid coordinating residues.

**Figure 9:**
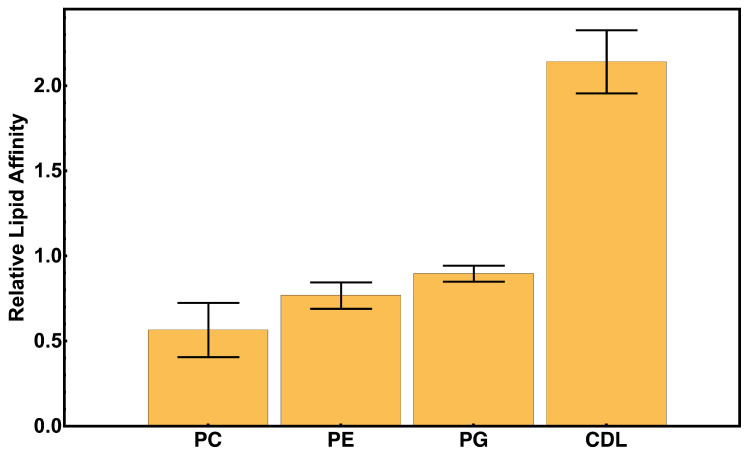
Lipid interaction survey. Relative lipid affinity is calculated as the fraction of the number of protein-bound lipids, normalized by percent composition for each lipid type. We observe a significant bias for cyt *c*_2_ to bind CDL.

Figure 8 shows the evolution of lipid contacts over a representative 200 ns simulation. The trend of increasing and decreasing the number of total lipid contacts suggests a transient association with the membrane during the diffusion. As expected, the majority of the binding occurs between the protein and negatively charged phospholipids PG and CDL (Figure 8 and Figure 9). However, it should be noted that there is a nearly equal amount of interactions with CDL as there is PG, despite the concentration of CDL being roughly three times less than that of PG; this could suggest a binding bias towards CDL as opposed to PG. The higher affinity for CDL compared to PG can be primarily attributed to the higher charge density in the former.

We calculated the relative binding affinity for each lipid type in the membrane as the fractional contribution to protein–lipid interactions, normalized by the percent composition for 10 independent replicates (Figure 9). These measurements reveal a significant bias for cyt *c*_2_ to bind CDL.

**Figure 10:**
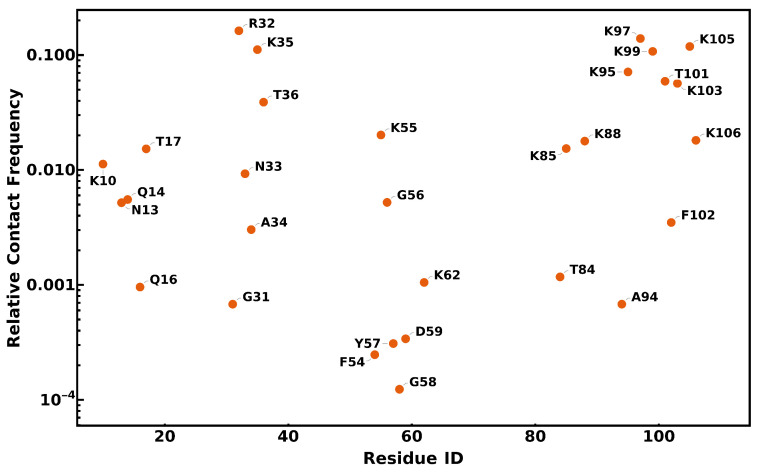
Cytochrome *c*_2_–lipid interactions. The relative contribution per cyt *c*_2_ residue towards the total number of lipid–protein interactions is shown. The criterion for a contact is defined as being within 2.5 Å of a cyt *c*_2_ heavy atom.

To determine which residues are most likely to interact with the membrane during diffusion, we measured the relative interaction frequencies for each protein residue (Figure 10). The interaction frequencies are typically higher for positively charged residues corresponding to the protein sequence (PDB 1L9B, Chain C). Residues with the higher interaction frequencies show similarities to those which coordinate with the reaction center, as presented in Axelrod et al., 2002 [9].

**Figure 11:**
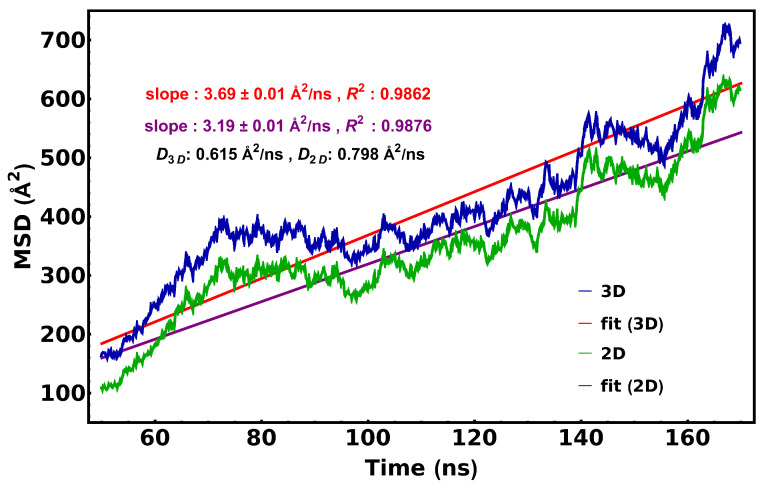
Diffusivity of cytochrome *c*_2_. The diffusion coefficient for cyt *c*_2_ was calculated from a linear fit to MSD vs. time plots obtained through an MD simulation (n = 10). Separate fits were performed, assuming three- and two-dimensional modes of diffusion.

A plot of the mean square distance (MSD) vs. time was used to determine the diffusion coefficient of cyt *c*_2_ (Figure 11). The fit obtained through linear regression yields a slope of 3.69 ± 0.01 Å^2^/ns for 3D diffusion, and a slope of 3.19 ± 0.01 Å^2^/ns for 2D diffusion. This is equivalent to a 3D diffusion constant of 0.615 Å^2^/ns or a 2D diffusion constant of 0.798 Å^2^/ns. These values are closer to the magnitude of typical lipid diffusion coefficients [39] than they are to what has been reported for other electron shuttle proteins [40,41].

## 4. Discussion

Previous studies have proposed a two-dimensional or restrictive type of diffusion on the surface of the membrane for members of the cytochrome family as they shuttle electrons across the ETC [19,20,42]. Additional studies based on oxidative kinetics have suggested that the mode of diffusion was mostly two-dimensional but could not rule out three-dimensional ones [21]. Our simulations suggest a “skipping” mode of diffusion for cyt *c*_2_ during diffusion between ETC components on a membrane containing negative lipids. During diffusion, cyt *c*_2_ maintains a stable tilt with respect to the membrane normal.

We find that the interactions between cyt *c*_2_ and the lipid bilayer are biased towards the anionic lipids, with a preference towards CDL. This is consistent with previous studies carried out by the Overfield Lab, which have suggested that anionic lipids can restrict the diffusion of cytochromes [19,20]. Furthermore, our simulations suggest that anionic lipid-induced diffusion restriction can still be observed in membranes of mixed composition. We speculate that this is owed in part to the high relative affinity for CDL compared to other lipids.

As expected, we observed that positively charged residues on the binding face of cyt *c*_2_ interact with the membrane the most. It is interesting to note that the same residues which interact the most with the lipid bilayer also show the most propensity for its binding with other proteins [3,9]. A similar pattern was observed for binding sites of cytochrome *c* with Complex III and Complex IV in the mitochondrial ETC [43,44]. Although we do see an increase in the measured diffusion constant across membranes with a decreasing concentration of anionic lipids, we do not recover diffusion coefficients comparable to those predicted by kinetics based estimates [20]. However, the measured diffusion coefficients from our MD simulations are in close agreement with reported values for cytochromes and cytochrome peroxidase, as observed in Brownian dynamics studies [45]. In this work, we have proposed a mode of diffusion for cytochromes in transit between ETC components.

A major limitation of this model is that it does not include the effects of other proteins that may be present in a physiological setting. However, experimental studies have suggested that the bilayer size chosen is comparable to what has been observed for the space between individual proteins which comprise respiratory supercomplexes [46]. Given the propensity of cytochromes to bind CDL and the role of CDL in mediating supercomplex assembly, exploring the effects of protein competition on diffusion mechanics is suggested as a future direction for this study. Nevertheless, the reported results in this study clearly suggest an additional role for negatively charged lipids in controlling the dynamics of bioenergetic membranes’ electron transfer reactions.

## Data Availability

Data supporting reported results can be found in https://zenodo.org/records/15200185 (accessed on 1 March 2025).

## Supplementary Materials

The following supporting information can be downloaded at: https://www.mdpi.com/article/10.3390/membranes15040124/s1.
